# Identification of molecular pathway changes after spinal cord injury by microarray analysis

**DOI:** 10.1186/s13018-016-0437-3

**Published:** 2016-09-15

**Authors:** Haocong Zhang, Yan Wang

**Affiliations:** Department of Orthopaedics, The General Hospital of PLA, No. 28 Fuxing Road, Haidian District, Beijing, 100853 China

**Keywords:** Differentially expressed genes, Interaction network, Pathway enrichment analysis, Spinal cord injury

## Abstract

**Background:**

Spinal cord injury (SCI) is highly related to the devastating sensory and motor dysfunction.

**Methods:**

The GSE45006 gene expression profile dataset was downloaded from Gene Expression Omnibus, which was collected from 24 rats including 20 animals with injured T7 spinal cords using an aneurysm clip impact-compression injury model and killed after 1 day, 3 days, 1 week, 2 weeks, and 8 weeks and four sham-operated rats. Differentially expressed genes (DEGs) between the injured rats at each time point and the sham-operated rats were screened. DEGs commonly detected throughout different time points were further identified, followed by comparing the expression level of these DEGs at each time point between the injured spinal cord samples and controls. Pathway enrichment analysis of the common DEGs was performed.

**Results:**

The difference in the expression level of 416 common DEGs was significant between the injured spinal cord samples and the controls at each time point (*P* < 0.05), with the most significant difference 1 day after SCI. The common DEGs were enriched in three pathways, namely Fcγ R-mediated phagocytosis, mitogen-activated protein kinase (MAPK) signaling pathway, and chemokine signaling pathway. *AKT3* and *RAC2* were enriched in all the three pathways; *RAP1B* in both MAPK signaling pathway and chemokine signaling pathway; and *VAV1*, *LYN*, and *HCK* in both Fcγ R-mediated phagocytosis and chemokine signaling pathway.

**Conclusions:**

This study has confirmed the occurrence of neuronal death, inflammation, and neuronal regeneration after SCI. *AKT3*, *RAC2*, *VAV1*, *RAP18*, *LYN*, and *HCK* may have critical roles in the pathological responses to SCI.

## Background

Spinal cord injury (SCI) can lead to sensory and motor dysfunction, manifesting a wide range of functional deficits depending on the level of injury [1, 2]. Following the mechanical injury that rapidly kills neurons and glia at the initial injury site, devastating and delayed secondary cellular and molecular events occur. These include enlarged cell death through necrosis and apoptosis, which is exacerbated by the presence of inflammatory cells, such as neutrophils, macrophages, and T cells [3, 4]. Among these inflammatory cells, macrophages, a type of phagocyte, have a dominant role, which not only damage neurons and glia but also are essential for the reconstruction of injured tissues by phagocytosis [5]. Chemokines, a type of small chemoattractant peptide, which can provide directional cues for the cell trafficking, are also necessary for the inflammatory responses [6]. Microarray analysis has been proven reliable and efficient in elucidating the molecular events following SCI [7–9]. For example, microarray analysis together with immunohistochemical studies has validated neuronal regeneration after SCI based upon the up-regulation of neurite growth and regeneration-associated genes after SCI, such as those encoding VGF, SPRR1A, and GAP-43 [10–12]. Chamankhah et al. have identified gene expression profiles at day 1, day 3, week 1, week 2, and week 8 in rats following SCI, and they have found that the adaptive and induced innate immune responses span the acute and subacute phases and also persist throughout the chronic phase of SCI, despite the induced innate responses are more active during the acute phase, while the adaptive immune response processes are more significant during the chronic phase [13].

In the present study, the GSE45006 microarray data submitted by Chamankhah et al. that were collected from rats at different time points after SCI were downloaded and further analyzed. Here, we identified differentially expressed genes (DEGs) that were common throughout different time points. Then, based on the common DEGs, pathway enrichment analysis was performed and an interaction network was also constructed. These common DEGs may be essential for the pathological responses in rats after SCI, thus can be alternatives for screening new therapeutic targets for spinal cord-injured patients.

## Methods

### Microarray data

Gene expression profile dataset GSE45006 was downloaded from the Gene Expression Omnibus (GEO, http://www.ncbi.nlm.nih.gov/geo/) database. The annotation platform was GPL1355 [Rat230_2] Affymetrix Rat Genome 230 2.0 Array. As described by Chamankhah et al., a total of 24 animals were used and divided into six groups of four animals each. Twenty female Wistar rats in five groups underwent a T6-T8 laminectomy and then received a 35-g (Walsh) moderate to severe aneurysm clip impact-compression injury at T7 for 1 min, which were killed after 1 day, 3 days, 1 week, 2 weeks, and 8 weeks following the surgery. For each operated animal, a tissue sample was collected from the T7 spinal cord epicenter for extraction of total RNA; meanwhile, the samples were also collected from the T7 spinal cord tissue of sham-operated rats in the control group.

### Microarray data preprocessing and screening of DEGs

All the hybridized probes were submitted to the annotation platform to obtain gene names, and null probes were removed. Then, the expression profile data of the resulting genes were subjected to log2 conversion [14].

Genes with |log2FC (fold change)| >1 and a false discovery rate (FDR) value <0.05 were selected as DEGs [15]. The FDR value was obtained by adjusting the raw *P* values with the BH (Benjamin–Hochberg) method [16].

### Further comparison of common DEGs

The common DEGs throughout different time points were further screened, which were defined as successive DEGs. Then, *t* test was used to determine whether there was significant difference in the expression level of these common DEGs between the injured spinal cord samples and the controls at each time point (*P* < 0.05) [17].

### Pathway enrichment analysis of the common DEGs

Database for Annotation, Visualization and Integrated Discovery (DAVID, https://david.ncifcrf.gov/home.jsp) bioinformatics resources consist of an integrated biological knowledgebase and analytic tools aimed at systematically extracting biological meaning from large gene or protein lists [18]. The Gene Functional Classification Tool of DAVID was used to perform pathway enrichment analysis based on the Kyoto Encyclopedia of Genes and Genomes (KEGG) database using a gene-set approach to determine whether there is a functional change of a set of genes (*P* < 0.05) [19, 20].

### Construction of interaction network based on pathway

Search Tool for the Retrieval of Interacting Genes/Proteins (STRING) can provide uniquely comprehensive coverage and ease of access to both experimental as well as predicted interaction information that is denoted with a confidence score [21]. In the present study, STRING was used to construct an interaction network of the DEGs significantly enriched in pathways, and a score threshold of 0.4 was adopted. The resulting network was visualized using Cytoscape [22].

## Results

### DEGs screened at each time point after SCI

In total, 19,763 genes were obtained for DEG screening after removing null probes. A total of 3405, 2165, 1262, 1391, and 1841 DEGs were obtained at day 1, day 3, week 1, week 2, and week 8 following SCI, respectively.

### Further comparison of the expression of the common DEGs

**Fig. 1:**
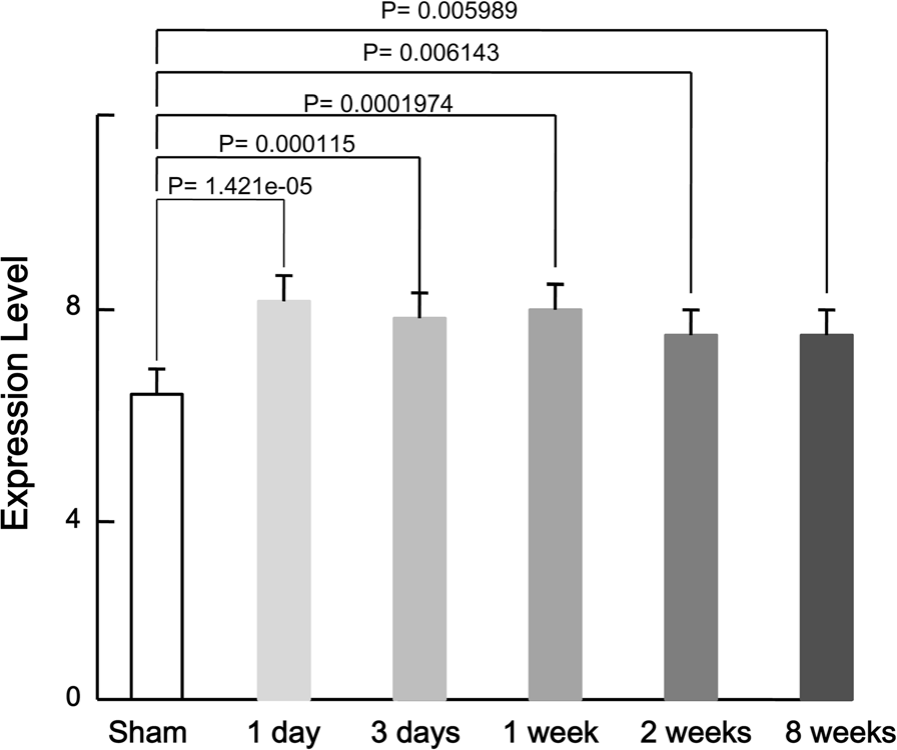
Further comparison of the expression levels of the common DEGs between the injured spinal cord samples at 1 day, 3 days, 1 week, 2 weeks, and 8 weeks after SCI and the control successively

A total of 416 DEGs were found common throughout the five different time points, such as the up-regulated ones, *RAC2*, *RAP1B*, *VAV1*, *LYN*, *HCK*, *JUN*, and the down-regulated ones, *AKT3* (Table 1). The result of the *t* test revealed that there was significant difference in the expression level of these common DEGs between the injured rats at each time point and the sham-operated ones (*P* < 0.05), with the most significant difference 1 day after SCI (*P* = 1.42e-05), followed by less significant difference, 3 days (*P* = 0.000115), 1 week (*P* = 0.0001974), 8 weeks (*P* = 0.006143), and 2 weeks (*P* = 0.005989), after SCI successively (Fig. 1).

**Table 1:** Some of the genes successively differentially expressed across different time points

	On day 1		On day 3		At week 1		At week 2		At week 8	
Gene symbol	logFC	adj. P val	logFC	adj. P val	logFC	adj. P val	logFC	adj. P val	logFC	adj. P val
Akt3	−2.9097	0.000187	−1.96743	0.000164	−2.19211	0.002497	−2.92584	1.42E-06	−2.42503	6.64E-05
Jun	1.524331	0.000472	1.83288	0.00021	1.795763	0.002161	0.002161	1.795763	1.092874	0.00173
Rap1b	1.592105	0.000158	1.131165	0.00048	1.546159	0.004442	1.2095	3.48E-05	1.285494	0.000403
Lyn	4.070571	0.000472	2.399984	0.00572	3.612514	0.006097	3.195225	0.000343	2.950347	0.00239
Rac2	4.305635	0.000242	2.226036	0.00487	3.706749	0.005252	2.863293	0.000298	2.376156	0.00531
Vav1	5.353884	3.86E-05	2.458724	0.00195	4.618522	0.003165	3.970052	1.08E-05	4.247292	8.95E-05
Hck	7.363526	5.02E-06	5.202298	4.22E-05	6.446867	0.001662	5.930991	7.95E-07	6.162797	1.23E-05

### Pathway enrichment analysis of the common DEGs

According to the pathway enrichment analysis, the 416 common DEGs were enriched in three pathways (Table 1), that is, Fcγ R-mediated phagocytosis pathway (*P* = 4.367e-04), mitogen-activated protein kinase (MAPK) signaling pathway (*P* = 0.001434), and chemokine signaling pathway (*P* = 0.001592).

### Interaction of DEGs significantly enriched in the three pathways

**Fig. 2:**
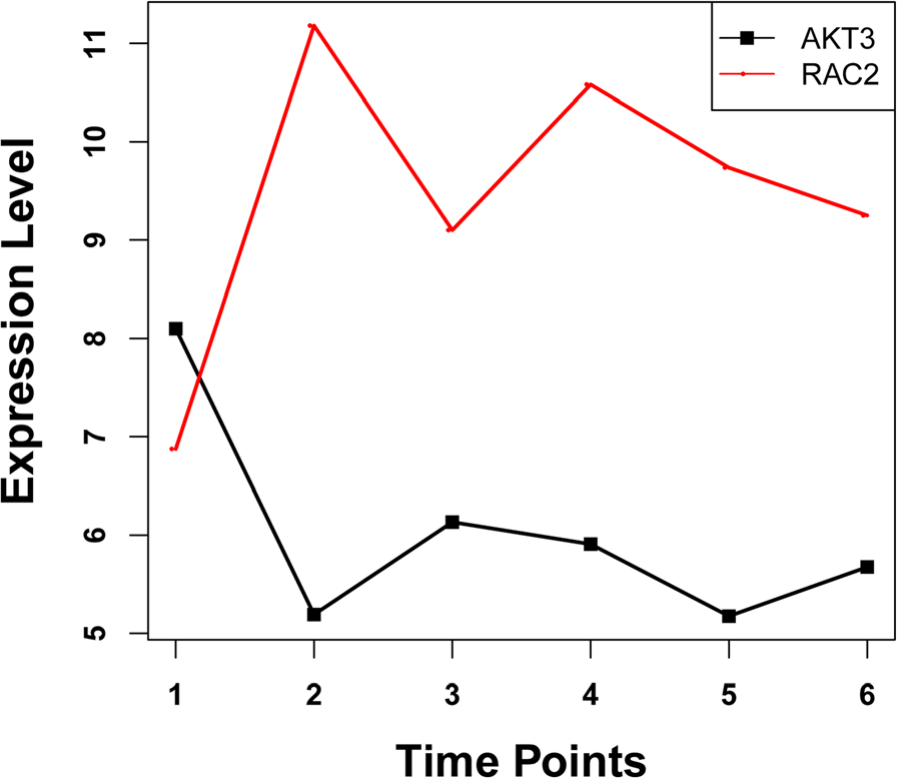
The expression profile of *AKT3* and *RAC2* over time after spinal cord injury. The numbers 2, 3, 4, 5, and 6 on the *horizontal ordinate* represent 1 day, 3 days, 1 week, 2 weeks, and 8 weeks after SCI, respectively, and the *first point* of each *line* represents the expression level of the corresponding gene in the control

**Fig. 3:**
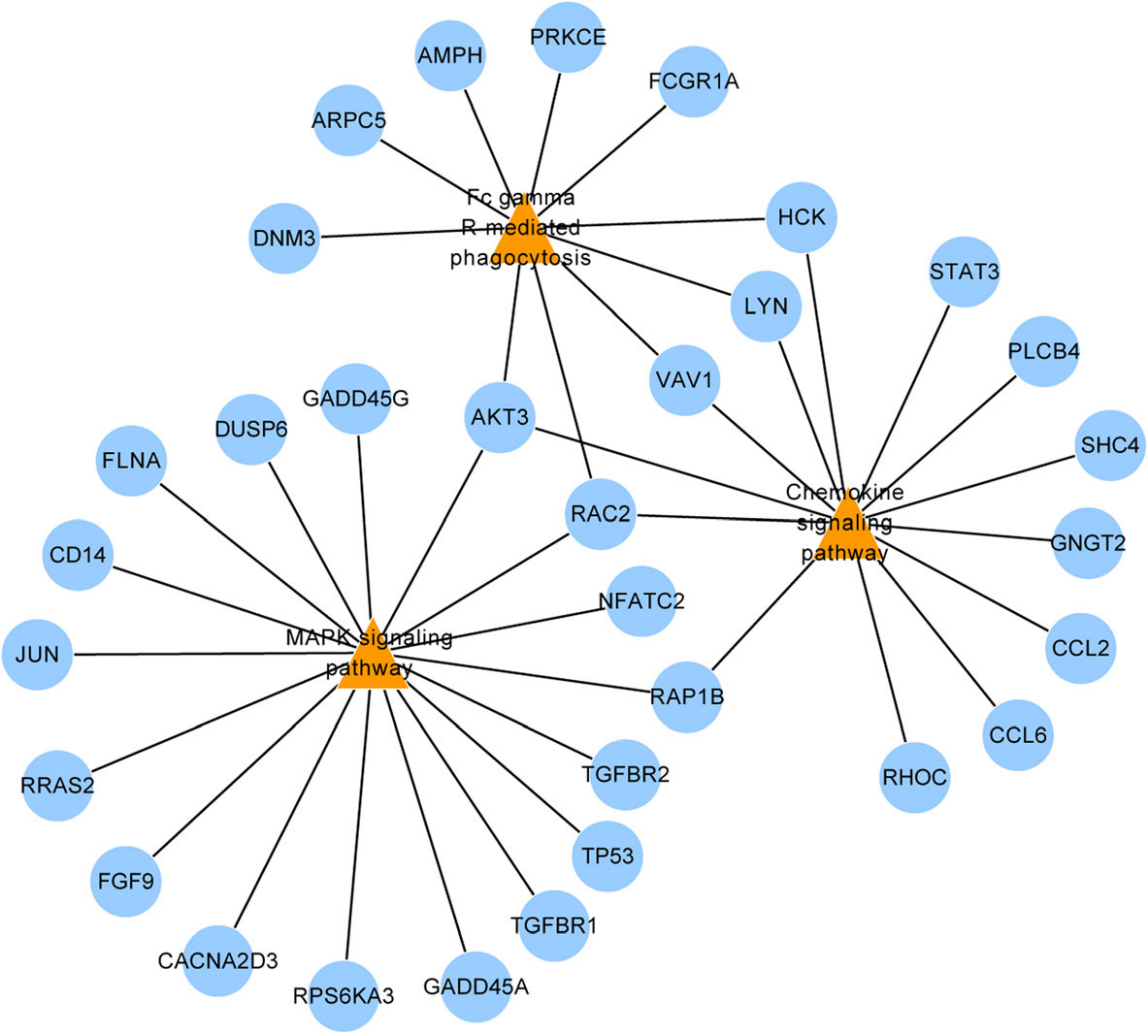
Interaction of DEGs significantly enriched in the three pathways. The *orange triangles* represent the three enriched pathways; the *blue circles* represent the DEGs enriched in the pathways

According to the constructed interaction network of DEGs significantly enriched in the three pathways, *AKT3* (serine/threonine protein kinase 3) and *RAC2* (Ras-related C3 botulinum toxin substrate 2) were enriched in all the three pathways (Fig. 2); *Rap1B* (Ras-related protein RAP1B) was involved in both the MAPK signaling pathway and the chemokine signaling pathway; *VAV1* (guanine nucleotide exchange factor) and two Src family members, *LYN* (tyrosine protein kinase) and *HCK* (hemopoietic cell kinase), were involved in both the Fcγ R-mediated phagocytosis and the chemokine signaling pathway (Fig. 3).

## Discussion

Chamankhah et al. have performed both time point and time-series analyses of the identified DEGs, which were further analyzed using Gene Ontology (GO) [13]. In the present study, after we identified genes differentially expressed at each time point, only those expressed successively across different time points were further studied by investigating the pathways these genes were involved in, through which it is found that the common DEGs were significantly enriched in three pathways, namely Fcγ R-mediated phagocytosis pathway, MAPK signaling pathway, and chemokine signaling pathway, and may have a profound implication for the pathological responses in rats at the cellular and molecular level after SCI. The chemokine signaling pathway is transduced by chemokine receptors (G protein-coupled receptors) expressed on the immune cells, which mediate the activation-diverse downstream pathways resulting in cellular polarization and actin reorganization [6]. The Fcγ R-mediated phagocytosis is mediated by the Fcγ receptors (Fc receptor for immunoglobulin G) [23]. Despite phagocytosis being universally acknowledged after SCI [5, 24], Fcγ R-mediated phagocytosis is seldom reported in spinal cord-injured rats. These two enrichment pathways further confirmed the key role of inflammation after SCI, conforming with numerous previous studies [3, 4, 25, 26] that have demonstrated the involvement of two major types of inflammatory cells within certain time periods after SCI. For the MAPK cascade pathway, it is a highly conserved module that is involved in various cellular functions. Two MAPKs, *ERK*1/2 (extracellular signal-related kinases) and p38 MAPK (p38alpha/beta/gamma/delta), mediate inducible nitric oxide synthase (iNOS)-induced spinal neuron degeneration, which is a general way to cause neuronal apoptosis by generating nitric oxide (NO) after acute traumatic SCI [27].

*AKT3* and *RAC2* were enriched in all the three pathways, indicating their important role after SCI. The AKT (also called protein kinase B) family consists of three members, Akt1/PKBα, Akt2/PKBβ, and Akt3/PKBγ, which share a high degree of structural similarity [28]. Peviani et al. have proposed that down-regulation or lack of induction of the P13K/AKT prosurvival pathway may be responsible for the defective response of spinal cord motor neurons to injury and their consequent cell death [29]. The finding that *AKT3* is significantly down-regulated across all the time points overall in our study further validates their proposal.

The Rho guanosine triphosphatases (GTPases) comprising RAC1 and RAC2 are key regulators of cytoskeletal dynamics and affect many cellular processes, including cell polarity, migration, vesicle trafficking, and cytokinesis [30]. Overall, the expression of *RAC*2 was up-regulated after SCI in our study, which is consistent with the finding that Rho activity is raised after SCI [31]. Activation of Rac GTPase has been proven to be involved in the p75 neurotrophin receptor-dependent apoptosis [31, 32]. Furthermore, Harrington et al. have reported that this apoptosis pattern is mediated by activation of c-jun N-terminal kinase [32], which can phosphorylate c-JUN, a major component of the JUN family protein (c-JUN, JUNB, and JUND) members that are necessary for the assembly of the AP-1 transcription factor complex [33]. c-JUN, involved in the MAPK signaling pathway, is highly induced in response to neuronal injury [34, 35], which is consistent with the up-regulation of its expression observed in our study.

Additionally, *VAV1*, *LYN*, and *HCK* were involved in both the Fcγ R-mediated phagocytosis and the chemokine signaling pathway. *VAV1* is engaged in the regulation of T cell migration [36], and the up-regulation of its expression proves the participation of T cells in immune response after SCI. The expression of two Src family members, *HCK* and *LYN*, with similar roles in a signal pathway, were both up-regulated in our study, which is consistent with the up-regulation of *LYN* observed by Yang et al., who also studied the peripheral nerve injury-induced modification of genes in Sprague-Dawley rat dorsal spinal cords using both microarray analysis and immunohistochemistry [9]. Meanwhile, *RAP1B* (Ras-related protein Rap-1B) was also observed to participate in both the MAPK signaling pathway and the chemokine signaling pathway. Schwamborn and Püschel have discovered that localization of the GTPase RAP1B (Ras-related protein Rap-1B) to the tip of a single neurite is a decisive step in determining which neurite becomes the axon [37]; thus, the up-regulation of RAP1B expression in our study may suggest the occurrence of neuronal regeneration after SCI.

## Conclusions

In summary, we identified genes that were differentially expressed at different time points after SCI in rats, as well as three pathways, Fcγ R-mediated phagocytosis pathway, MAPK signaling pathway, and chemokine signaling pathway, through which these DEGs were speculated to respond to SCI. This further proves that neuronal death, inflammation, and neuronal regeneration occur after SCI, especially inflammation. And, DEGs, *AKT3*, *RAC2*, *VAV1*, *RAP1B*, *HCK*, and *LYN*, that are involved in the above three pathways may be essential for the pathological responses to SCI, which may be considered as candidate genes for the targeted therapy of SCI. Our work has deepened our understandings into the molecular mechanisms underlying SCI. However, since our findings here were obtained by bioinformatics methods, they should be taken prudently and further validated by experimental proofs before any clinical use.

## Abbreviations

AKT3: Serine/threonine protein kinase 3
BH: Benjamin–Hochberg
DAVID: Database for Annotation, Visualization and Integrated Discovery
DEGs: Differentially expressed genes
FDR: False discovery rate
GEO: Gene Expression Omnibus
GO: Gene Ontology
GTPases: Guanosine triphosphatases
HCK: Hemopoietic cell kinase
iNOS: Inducible nitric oxide synthase
KEGG: Kyoto Encyclopedia of Genes and Genomes
LYN: Tyrosine protein kinase
NO: Nitric oxide
RAC2: Ras-related C3 botulinum toxin substrate 2
Rap1B: Ras-related protein RAP1B
RAP1B: Ras-related protein Rap-1B
SCI: Spinal cord injury
STRING: Search Tool for the Retrieval of Interacting Genes/Proteins
VAV1: Guanine nucleotide exchange factor
